# Supplementary figures and images for: Photosynthetic performance and stevioside concentration are improved by the arbuscular mycorrhizal symbiosis in Stevia rebaudiana under different phosphate concentrations
Source: PeerJ. 2020 Oct 19;8:e10173. doi: 10.7717/peerj.10173 (PMC7580585; doi:10.7717/peerj.10173)

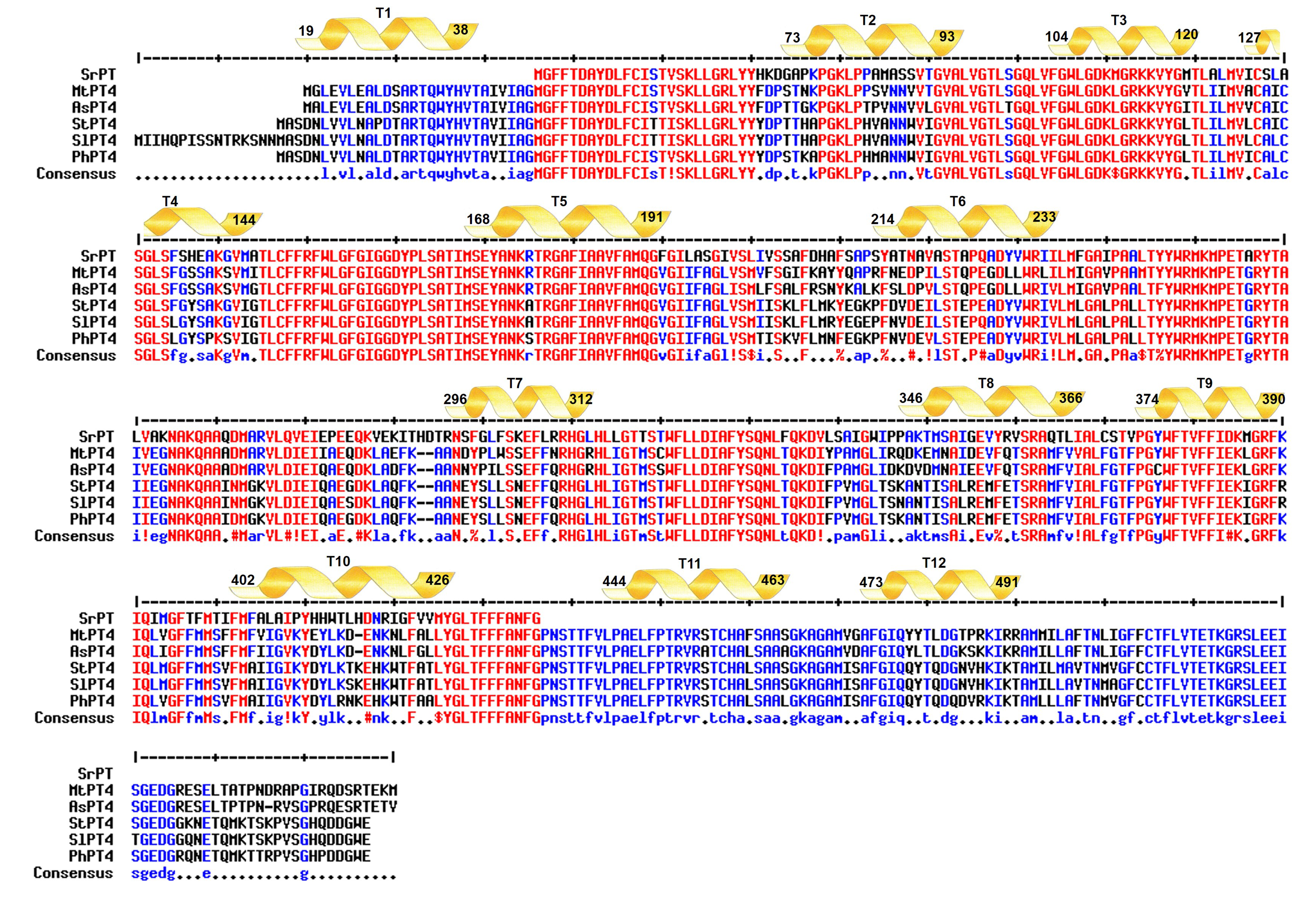

Supplement: Supplemental Information 1 — The SrPT protein contains 391 amino acids and 9 TM domains. The alignment that was used to deduce the amino acid sequence and topology of the 12 SrPT TM domains (TM1-TM12) was predicted using AM-specific phosphate transporters from M. truncatula (MtPT4), A. sinicus (AsPT4), S. tuberosum (StPT4), L. esculentum (LePT4), and P. hybrida (PhPT4). The deduced amino acid sequence was aligned through MULTIALIN, and SrPT was predicted according to Yadav et al. (2010). [file peerj-08-10173-s001.png]

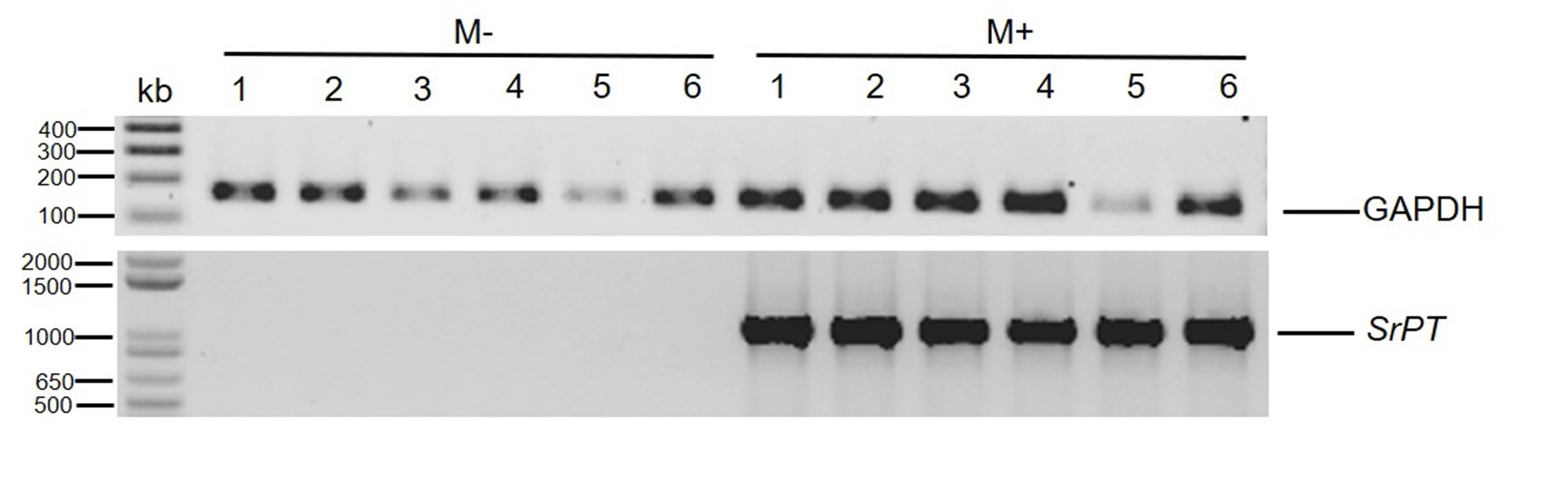

Supplement: Supplemental Information 2 — Lanes 1-6, replicates of noncolonized (M-) and R. irregularis-colonized plants (M+). SrGADPH was used as a reference gene. [file peerj-08-10173-s002.png]

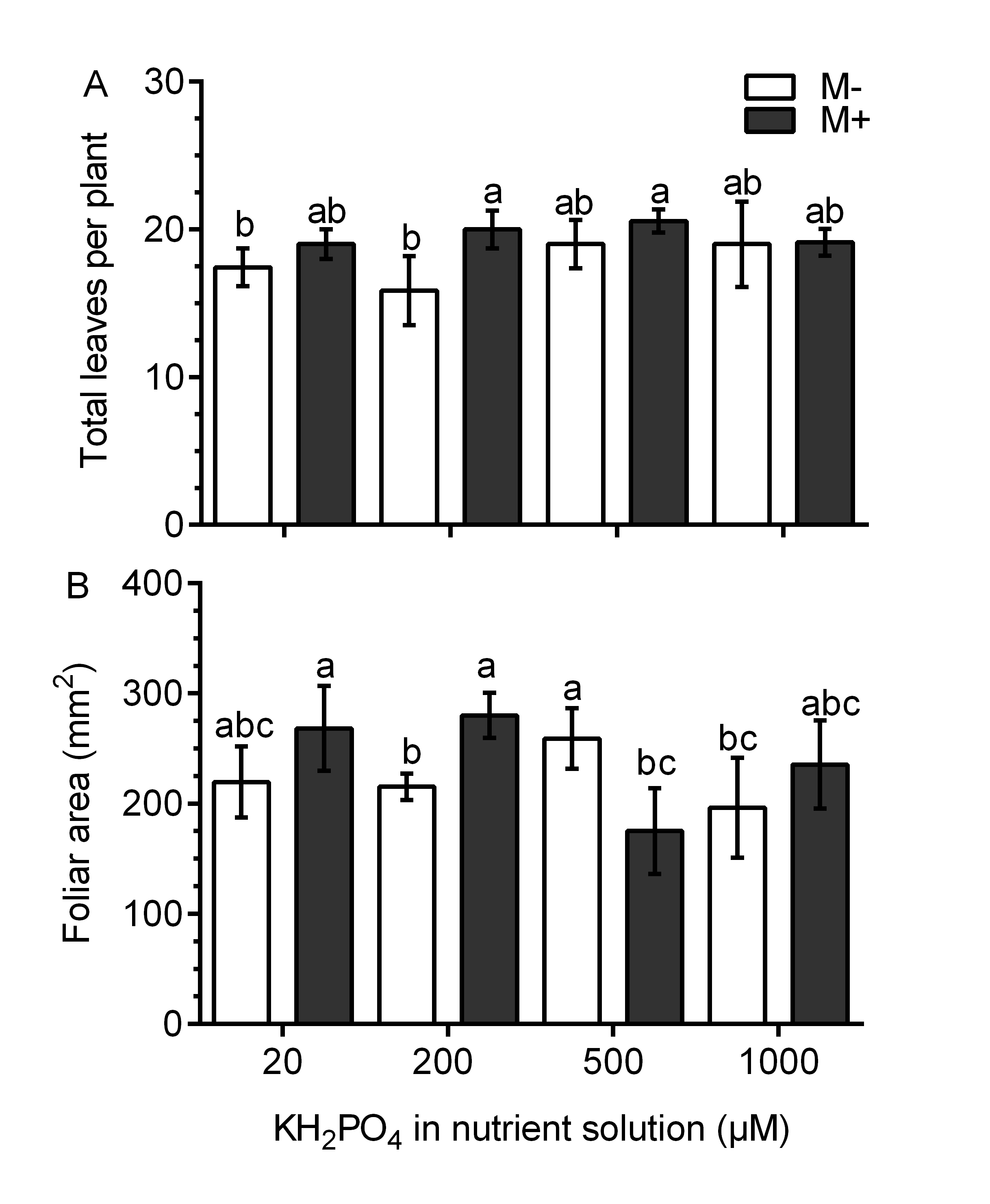

Supplement: Supplemental Information 3 — Leaves number (A) and foliar area in leaves of mycorrhiza-colonized (M+) and noncolonized (M-) S. rebaudiana plants fertilized with Hoagland solution using different KH2PO4 concentrations Bars represent the mean ±standard deviation (SD) of six replicates. Different letters indicate significant differences according to Tukey’s test ( P < 0.05). [file peerj-08-10173-s003.png]

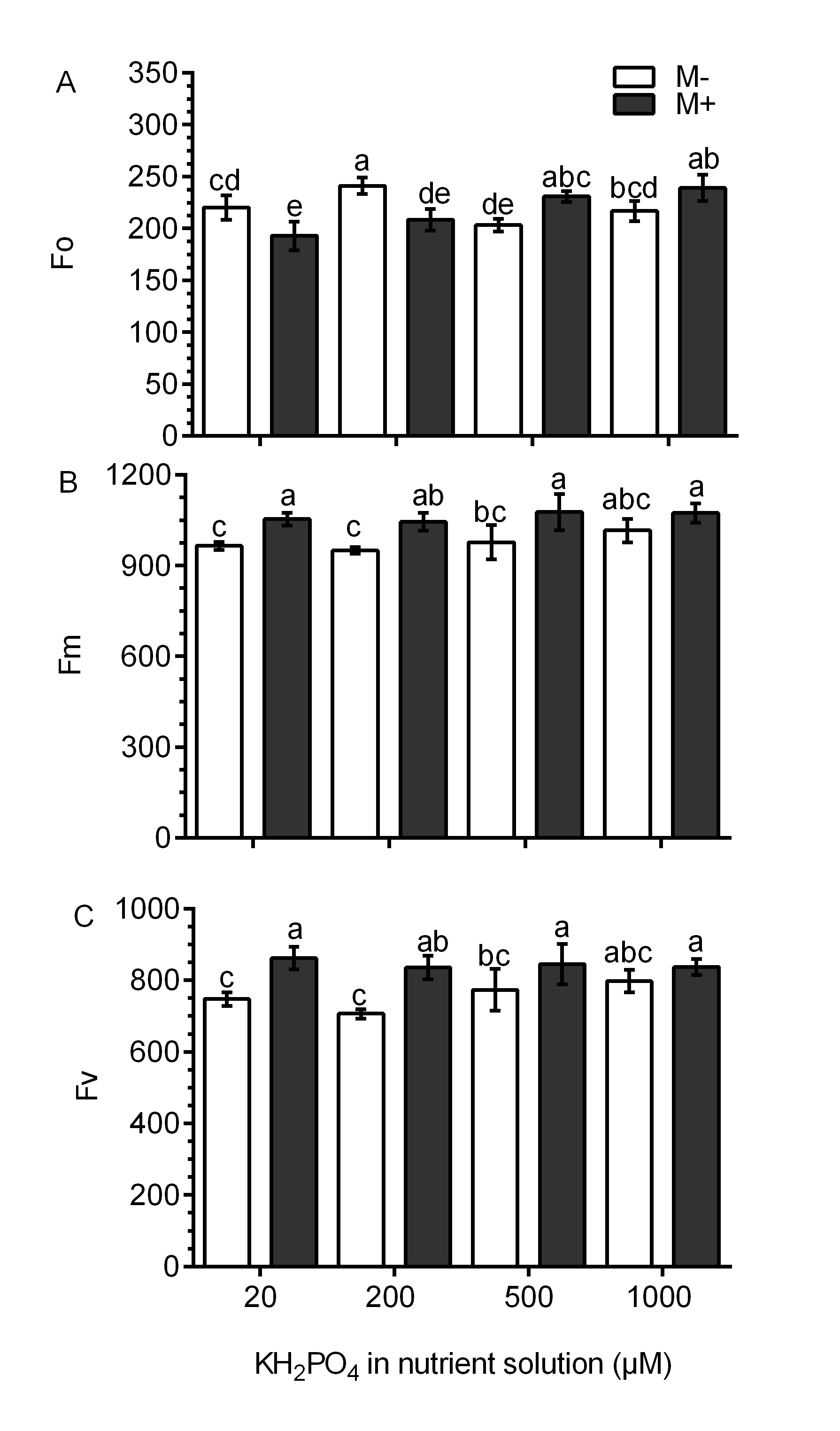

Supplement: Supplemental Information 4 — Primary fluorescence (Fo) (A), maximal fluorescence (Fm) (B), and variable fluorescence (Fv) (C) in leaves of mycorrhiza-colonized (M+) and noncolonized (M-) S. rebaudiana plants fertilized with Hoagland solution using different KH2PO4 concentrations. Bars represent the mean ±standard deviation (SD) of six replicates. Different letters indicate significant differences according to Tukey’s test ( P < 0.05). [file peerj-08-10173-s004.png]
